# Ulcerative Colitis: Current and Emerging Treatment Strategies

**DOI:** 10.3390/jcm9010094

**Published:** 2019-12-30

**Authors:** Maia Kayal, Shailja Shah

**Affiliations:** 1 Division of Gastroenterology, Icahn School of Medicine at Mount Sinai, New York, NY 10029, USA; Maia.Kayal@mountsinai.org; 2 Division of Gastroenterology, Hepatology and Nutrition, Vanderbilt University Medical Center, Nashville, TN 97203, USA

**Keywords:** ulcerative colitis, biologic, small molecule, colectomy

## Abstract

Historically, medical therapy for ulcerative colitis (UC) was limited to corticosteroids. Excitingly, over the past just 1–2 decades, the options for medical therapy have expanded and include biologics and small molecules, with more agents actively being developed. In this article, we review the current and emerging treatment strategies for UC stratified according to disease severity.

## 1. Introduction

Ulcerative colitis (UC) is a chronic inflammatory disorder defined by mucosal inflammation that involves the colon and rectum in a continuous pattern [1,2,3]. The peak age of onset is 30–40 years old, and men and women are affected equally [4]. While still not yet fully defined, the pathogenesis of UC is multifactorial and implicates environmental factors, aberrant host immune responses, and likely intestinal dysbiosis in genetically susceptible individuals [3]. The global burden of UC continues to rise, along with the associated healthcare and societal costs. In the US alone, the annual direct and indirect costs related to UC are estimated to be $8.1 billion–$14.9 billion [5]. Because UC is a chronic disease with no known preventative or curative interventions, save colectomy, therapy is most often lifelong. The natural course of UC includes periods of remission interspersed with periods of acute exacerbations or disease flares, which might require escalation of therapy, hospitalization, and, in severe cases, colectomy. The goal of treatment is to achieve disease remission and prevent disease-related complications such as infection, surgery, and neoplasia, as well as preserve patients’ quality of life.

### 1.1. Selection of Therapy

Every effort should always be made to ensure there is shared therapeutic decision making between physicians and patients. There are many factors to consider when discussing therapeutic options with patients diagnosed with UC, including both disease-related (e.g., disease extent, inflammation severity) and patient-related factors (e.g., preferences, cost, comorbidities). Unfortunately, we are not yet in an era where we can reliably predict individuals’ responses to specific medical therapies, for example, based on individual serum or tissue analyses.

**Figure 1:**
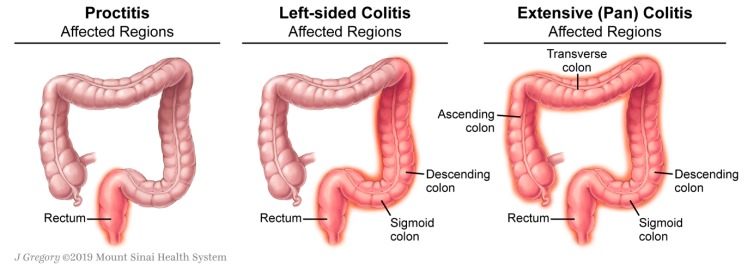
Ulcerative colitis disease extent.

The most important disease-related factors to consider include endoscopic/histologic and clinical disease severity as well as disease extent. Disease extent is defined as proctitis if inflammation is limited to the rectum, <15–20 cm from the anus. During their disease course, approximately 30% of adult patients with limited disease will have evidence of proximal extension based on endoscopy/histology or radiology. If mucosal involvement extends proximally from the rectum up to the splenic flexure (<50 cm from the anus) or past the splenic flexure, the disease is reclassified as either left-sided or extensive/pancolitis, respectively [2,6]. Limited proctitis occurs in 30–60% of adult patients with UC and manifests as hematochezia and tenesmus, left-sided colitis in 16–45% as proctitis plus diarrhea and abdominal cramping, and extensive colitis in 15–35% as left-sided colitis plus constitutional symptoms, fatigue, and fever (Figure 1) [3].

In all patients, triggering factors such as infection (e.g., *Clostridiodes difficile*, cytomegalovirus) should be evaluated for and managed appropriately. Appropriate treatment of infection should be initiated in conjunction with UC treatment in symptomatic patients with positive stool studies. These patients should be closely monitored after initiation of UC treatment as they may have a suboptimal response due to concomitant infection.

### 1.2. Goals of Therapy

In 2015, the Selecting Therapeutic Targets in Inflammatory Bowel Disease (STRIDE) committee defined the treat to target (T2T) approach in UC, which represented a paradigm shift away from treating primarily to clinical resolution of symptoms toward a more rigorous target of *additionally* treating to endoscopic/histologic remission, or so-called “mucosal healing”. Indeed, this shift was based on evidence demonstrating that mucosal healing is associated with long-term clinical remission, corticosteroid-free clinical remission, and avoidance of colectomy [7]. Adequate control of inflammatory burden over time also reduces the risk of colorectal neoplasia. Accordingly, the target for UC therapy is clinical remission defined as the resolution of rectal bleeding and diarrhea, *and* endoscopic remission defined as a Mayo endoscopic subscore of 0 or 1 [8].

Historically, medical therapy for UC was limited to corticosteroids. Excitingly, over just the past 1–2 decades, the medical therapeutic armamentarium now approved for the management of UC has exploded, and continues to expand. Clinical and endoscopic remission in UC may be achieved with several classes of medication including mesalamine, immunomodulators, corticosteroids, biologics and, most recently, small molecules. As stated above, the choice of therapy depends on multiple factors such as disease severity and extent, patient preference and expectations, medication formulation, and route of administration. Optimal management of UC requires an ongoing, close collaboration between patient and physician with shared decision making and informed consent. Herein, we review the current and emerging treatment strategies for adult patients with UC stratified according to disease severity. Medical management of extraintestinal manifestations including primary sclerosing cholangitis and complications of UC or therapy is outside of the scope of this review.

## 2. Mild-Moderate Ulcerative Colitis

Mild-moderate UC is defined clinically as <4–6 bowel movements per day with mild-moderate rectal bleeding in the absence of constitutional signs or symptoms such as fever and tachycardia, and laboratory abnormalities including elevated inflammatory markers and anemia [9,10]. Mild-moderate UC is defined endoscopically as mucosal erythema, decreased or absent vascularization, friability, and erosions [11].

Mesalamines are the first-line therapy for induction of remission in mild-moderate UC. There are different formulations of mesalamines, including oral, suppository, or liquid enema (Table 1). Selection among mesalamine formulations for treatment of mild-moderate UC depends primarily on disease extent. Indeed, based on a meta-analysis of 17 studies evaluating 2925 patients with mild-moderate UC on mesalamine therapy, there was no significant difference in the efficacy or safety of different mesalamine formulations [12]. Proctitis is managed with mesalamine suppository 1 g/day to target the involved rectum. Suppositories should be self-administered at bedtime and retained for 1–3 h for maximal benefit. Left-sided UC is managed with oral mesalamine 2–3 g/day and topical mesalamine 4 g/day enema formulation, which will reach the splenic flexure with appropriate use. Enemas should be administered at bedtime and retained overnight for approximately eight hours. Extensive mild-moderate UC is managed with oral mesalamine 2–3 g/day and topical mesalamine in either enema 4 g/day or suppository 1 g/day formulation. Clinical response is typically high, with 40–70% of patients expected to respond within 14 days; however, it can take up to eight weeks to achieve clinical and endoscopic remission [13,14]. In patients with prominent arthritic symptoms, sulfasalazine is an acceptable alternative to mesalamine, though often poorly tolerated due to side effects such as headache, nausea, diarrhea, and rash [9].

Second-line therapies for patients with mild-moderate UC who do not respond to mesalamine are corticosteroids. Systemic corticosteroids and budesonide-multimatrix (MMX) are both effective in induction of remission; however, the latter formulation has the important benefit of minimal systemic absorption due to high first-pass hepatic metabolism and, thus, more favorable side effect profile [15,16,17]. In a placebo-controlled randomized clinical trial (RCT) of 510 patients with mild-moderate UC and inadequate response to mesalamine, 13% of patients randomized to budesonide-MMX reached the primary endpoint of combined endoscopic and clinical remission at eight weeks compared to 7.5% of patients randomized to placebo [18]. Patients typically demonstrate clinical response within seven to 10 days. Budesonide-MMX is dosed as 9 mg daily for six to 10 weeks for induction of remission. In patients who respond, the dose is tapered to 9 mg every other day for two weeks followed by discontinuation, for a total of eight to 12 weeks of therapy. If patients do not show initial response to budesonide-MMX, then systemic corticosteroids, namely prednisone, is an option to induce remission. Prednisone is started at 40 mg per day and clinical response should be expected within 1–2 weeks. After two weeks, the dose should be tapered by 5–10 mg per week [19]. Rectal steroids are available in suppository and liquid or foam enema formulations and are effective in induction of remission with a relative risk of 0.73 when compared to placebo [20,21,22]. Corticosteroids in any formulation are not indicated for maintenance of remission due to side effects of therapy, which are most pronounced with systemic corticosteroids and include mood disturbance, hyperglycemia, weight gain, acne, insomnia, avascular necrosis, and skin atrophy, among others.

Rectal mesalamine is superior to rectal corticosteroids for induction of remission [9]. In a meta-analysis of 13 trials comparing rectal mesalamine and rectal corticosteroids, topical mesalamine (enema formulation 1–4 g/day or suppository formulation 1 g/day) was superior to topical corticosteroids for inducing remission. [9] Given this, in addition to the potential safety concerns with long-term rectal corticosteroids, rectal mesalamine is preferred for mild-moderate UC. However, patients may prefer corticosteroid foam enemas to mesalamine liquid enemas because of ease of delivery and retention [23,24].

Patients who achieve remission with mesalamine therapy should continue on the same medication [13]. Steroids are not appropriate for maintenance of remission due to adverse effects and lack of long-term efficacy.

## 3. Moderate-Severe Ulcerative Colitis

Moderate-severe UC is clinically defined as 4–6 bowel movements per day with moderate-severe rectal bleeding in the absence of constitutional signs or symptoms [10]. Moderate-severe UC is defined endoscopically as marked mucosal erythema, absent vascularization, friability, granularity, spontaneous bleeding, and ulcerations [11].

As of this writing, agents currently approved for the induction and maintenance of remission of moderate-severe UC include the biologics infliximab, adalimumab, golimumab, vedolizumab, and ustekinumab, in addition to the small-molecule Janus kinase (JAK) inhibitor tofacitinib (Table 2) [25,26,27,28,29,30]. Generally speaking, prior to starting these agents and immunomodulators, all patients should have appropriate pre-initiation safety labs and vaccinations, although the latter are sometimes not possible due to acute presentation, as well as ongoing interval surveillance of healthcare maintenance needs.

Infliximab, adalimumab, and golimumab are monoclonal antibodies that target tumor necrosis factor (TNF)-alpha, an inflammatory cytokine that mediates intestinal tract inflammation and is increased in patients with active UC. In a meta-analysis of six studies including 1823 patients with moderate-severe UC, patients treated with anti-TNF agents were 2.5-fold more likely to achieve clinical remission compared to patients treated with placebo (relative risk 2.45, 95% CI: 1.72–3.47); no single agent was clinically superior to the others [31]. The expected time to clinical response after initiation of these agents ranged from one to eight weeks [32]. Infliximab is administered intravenously, while adalimumab and golimumab are administered subcutaneously. Schedules for induction and maintenance vary according to the agent, and might also be altered based on disease trajectory and response. Biosimilars are near-identical copies of biologic agents that are equivalent to originator agents in efficacy and safety. Biosimilars of infliximab and adalimumab have been approved for the management of moderate-severe UC and are increasingly being used due to their significantly reduced cost. Therapeutic drug monitoring is beyond the scope of this article, but is increasingly incorporated into clinical practice with the most robust data available for infliximab.

The combination of infliximab and azathioprine is superior in the achievement of corticosteroid-free remission than infliximab or azathioprine monotherapy alone [33]. In a trial of 239 patients with moderate-severe UC previously naïve to TNF inhibitors, patients who received infliximab and azathioprine experienced higher rates of corticosteroid-free clinical remission at 16 weeks compared with patients who received either infliximab or azathioprine alone [33]. The decision of combination therapy, however, must consider patient- and disease-related factors, a full discussion of which is beyond the scope of this review. Notably, there is no incremental benefit in continuing mesalamine therapy in patients with moderate-severe UC who are escalated to anti-TNF therapy [34].

Vedolizumab is a humanized monoclonal antibody that recognizes the α_4_β_7_ cell surface glycoprotein expressed on circulating B and T lymphocytes and selectively blocks gut lymphocyte trafficking [35]. In a meta-analysis of four studies including 606 patients with moderate-severe UC, vedolizumab was superior to placebo for induction of clinical and endoscopic remission [36]. Vedolizumab is administered intravenously in an induction and then maintenance phase, with patients typically demonstrating clinical response within six weeks of the first dose [30]. In the only head-to-head trial of biologic agents in patients with moderate-severe UC, vedolizumab was superior to adalimumab with respect to clinical remission and endoscopic improvement [37]. Vedolizumab has a more favorable side effect profile compared to the anti-TNF inhibitors given its gut selectivity, and is not significantly associated with an increased risk of serious infection or malignancy [36].

Ustekinumab, a monoclonal antibody directed against the p40 subunit of interleukin-12 and interleukin-23, is the newest biologic approved for moderate-severe UC. In a randomized, placebo-controlled trial of 961 patients with moderate-severe UC, patients treated with ustekinumab had significantly higher rates of clinical remission and endoscopic improvement at week eight compared to placebo [29]. Although the induction dose is administered intravenously as a one-time dose, the subsequent maintenance doses are administered subcutaneously, and might be more appealing for some patients. Clinical response is expected within three to six weeks of induction [29]. Similar to vedolizumab, ustekinumab offers a favorable infectious safety profile compared to the anti-TNF agents. The rates of serious adverse events in randomized clinical trials were equivalent in the ustekinumab and placebo groups [38].

Tofacitinib is a small-molecule JAK inhibitor that modulates interleukin signaling, blocks the downstream effects of proinflammatory cytokines, and is approved for patients with moderate-severe UC who have failed or cannot tolerate TNF inhibitors. Tofacitinib is an oral medication with a rapid onset of action; clinical response to induction dosing is typically experienced within three days [39]. Depending on disease and patient factors, induction dose ranges from 5 mg twice daily to 10 mg twice daily. In two randomized, placebo-controlled trials of 598 and 541 patients with moderate-severe UC, patients treated with tofacitinib 10 mg orally twice daily had higher rates of clinical and endoscopic remission at week eight compared to placebo [40]. Tofacitinib is associated with an increased risk of herpes zoster virus reactivation in patients with UC, thromboembolic events, and elevated lipid profiles [41]. The increased risk of thrombotic events is associated with the 10 mg, twice daily dosage, typically used for patients with UC refractory to anti-TNF agents. Individual thrombosis risk assessment should be performed for patients with UC with a history of thromboembolic disease or cardiovascular disease before tofacitinib is considered.

## 4. Acute Severe Ulcerative Colitis

Acute severe ulcerative colitis (ASUC) is defined as the presence of ≥6 bloody bowel movements per day plus tachycardia >90 bpm, fever >37.8 °C, hemoglobin <10.5 gm/dL, or erythrocyte sedimentation rate (ESR) >30 mm/h [10]. ASUC is a life-threatening condition for which hospitalization is required. Patients are at risk for bowel perforation, toxic megacolon, or colectomy.

Currently approved medical therapies for patients hospitalized with ASUC are steroids, infliximab, and cyclosporine. Care of hospitalized patients with ASUC involves a multidisciplinary approach with gastroenterology, medicine, and surgery teams, given the risk of significant morbidity and mortality [42]. The immediate goal of therapy is hemodynamic stability and clinical improvement. Patients should be counseled at the outset regarding expectations of medical therapy and that total colectomy might ultimately be indicated. Initial workup includes history, examination, appropriate lab workup, including infectious workup if indicated, endoscopic evaluation, and possibly imaging depending on the clinical scenario.

Systemic steroids administered as methylprednisolone 20 mg intravenously every eight hours, or equivalent, are still the mainstay as the initial therapy for hospitalized patients with ASUC. Approximately 65% of patients will have symptomatic response, typically within three to five days of steroid initiation [43]. Patients with no improvement after five days of systemic steroids are unlikely to respond, and inpatient escalation to infliximab or cyclosporine should be considered if medical management is still deemed appropriate [44,45]. In the absence of enteric infection, antibiotics are not indicated. 

As discussed above, infliximab is a TNF inhibitor with a rapid onset of action. Patients with ASUC typically experience clinical improvement with less stool frequency, less hematochezia, and decreased inflammatory markers within three to five days of infliximab initiation. Colectomy rates are significantly lower in hospitalized patients with ASUC treated with infliximab compared to those treated with immunomodulators, mesalamines, or no therapy [46]. In a randomized, placebo-controlled trial in 45 patients with severe UC, patients who received infliximab had significantly lower rates of colectomy or death at three months [25]. There are mixed observational data regarding the optimal dosing of infliximab for ASUC, specifically among patients with objective evidence of a particularly high inflammatory burden. Some data support higher upfront dosing with 10 mg/kg instead of the standard induction dose of 5 mg/kg while other data support an accelerated dosing regimen [47,48,49]. While we eagerly await prospective randomized, controlled trials to inform dosing of infliximab for ASUC, both patient- and disease-related factors must be considered when deciding on dosing regimen. Patients who respond to infliximab during admission should continue standard maintenance dosing.

Cyclosporine directly inhibits calcineurin, a component of cytokine gene transcription, and downregulates IL-2, IL-3, IL-4, and TNF-alpha. In a randomized, placebo-controlled trial of 11 patients with ASUC, 82% of patients treated with cyclosporine had clinical response within seven days [50]. Cyclosporine is administered as a continuous intravenous infusion for hospitalized patients with ASUC with close monitoring of levels every two days to achieve target concentrations [51]. Clinical response is typically seen within two to three days, and colectomy rates have been shown to be less in patients treated with cyclosporine [50,52]. Patients who have improvement of stool frequency to <6 bowel movements per day and resolution of hematochezia may be converted from intravenous to oral cyclosporine to be continued for three months [53]. Cyclosporine, while itself not appropriate for maintenance therapy, is an effective bridge to an alternative medication that is approved for UC maintenance. For example, cyclosporine in the acute hospitalized setting as a bridge to vedolizumab in the outpatient setting is one therapeutic approach [54].

The decision to escalate to infliximab or cyclosporine depends on patient co-morbidities, physician experience, insurance considerations, and patient preference. For example, patients with renal disease, hypertension, history of seizures, or low serum cholesterol are not appropriate candidates for cyclosporine. The efficacy and safety profiles are not significantly different between infliximab and cyclosporine for patients with ASUC refractory to systemic steroids [55,56,57,58]. In a randomized controlled trial of cyclosporine vs. infliximab in patients with ASUC, treatment failure as defined by absence of clinical response at day 7 occurred in 60% of patients on cyclosporine and 54% of patients on infliximab (*p* = 0.52) [55]. Patients with ASUC who do not respond to infliximab or cyclosporine should be evaluated for inpatient colectomy [45,59].

Tacrolimus, a calcineurin inhibitor considered to be more potent than cyclosporine, is infrequently used for the management of adult patients with ASUC. Small observational studies in children with ASUC note comparable efficacy of oral tacrolimus (0.2 mg/kg per day in two divided doses) with intravenous cyclosporine in achieving short-term clinical improvement and reduction of pediatric disease activity assessment scores [60,61]. Additional studies regarding the use of tacrolimus in adult patients with UC are needed before formal recommendations are made.

## 5. Surgery

The most common surgery performed for patients with medically refractory UC but without complications, such as perforation, is the restorative proctocolectomy (RPC) with ileal pouch anal anastomosis (IPAA). This continence-preserving procedure involves the complete removal of the colon and rectum with construction of a ‘J’ shaped pouch from the terminal ileum to serve as an internal pelvic reservoir for intestinal contents. RPC with IPAA is typically performed in three stages: Stage 1 is the removal of the colon and creation of an end ileostomy, stage 2 is the removal of the rectum and construction of the IPAA with a diverting ileostomy, and stage 3 is the reversal of ileostomy and restoration of intestinal continuity and fecal stream. TPC with IPAA is associated with improved quality of life; however, it may be complicated by inflammatory conditions such as acute and chronic pouchitis.

## 6. Therapies with Limited Evidence

Curcumin has immunomodulatory and pro-apoptotic properties and is well tolerated without significant harmful effects. Results from a meta-analysis of six randomized controlled trials of 349 patients with mild-moderate UC on standard dose mesalamine suggest that adjuvant curcumin was effective in the induction of clinical remission, endoscopic remission, and endoscopic improvement, but not clinical improvement [62]. Due to limited evidence, society guidelines make no formal recommendations regarding the use of curcumin [9].

Probiotics have frequently been studied in patients with UC. In a meta-analysis of 22 studies examining the impact of probiotics on inflammatory bowel disease (IBD), there was no benefit of probiotics for induction of remission in patients with ulcerative colitis. However, when only studies of VSL#3 probiotic were included, there was a noted benefit in induction of remission (relative risk 0.74, 95% CI 0.63–0.87). Evidence regarding probiotic use in ulcerative colitis is limited due to small sample sizes, significant methodological heterogeneity, and risk of bias. Society guidelines recommend further study before use of probiotics [63].

The use of fecal microbiota transplant (FMT) for mild-moderate UC is considered experimental. Pooled analysis of RCTs that enrolled patients with mild-moderate UC noted that FMT was effective in the induction of clinical and endoscopic remission [64,65,66,67]. However, there was significant heterogeneity regarding donor stool, formulation, and administration schedule. Society guidelines recommend that FMT be performed only in the context of a clinical trial in patients with mild-moderate UC without *Clostridiodes difficile* at this time [9].

## 7. Emerging Therapies

The use of tofacitinib has been investigated in ASUC, given its rapid onset of action, its appropriateness and efficacy as both an induction and maintenance agent, and, relatedly, its safety profile. In a small retrospective series of four hospitalized patients with ASUC, high intensity tofacitinib dosed as 10 mg three times a day was associated with rapid improvement in clinical symptoms and inflammatory biomarkers [68]. These results suggest tofacitinib could be an effective therapeutic option for patients with ASUC who previously failed TNF inhibitors. However, additional clinical trial data including intervals for dose de-escalation are needed before formal clinical practice recommendations can be made.

Hyperbaric oxygen therapy has also been investigated in ASUC. The hypothesized mechanism of action is that pure excess oxygen delivery might reverse the tissue hypoxia that occurs in UC, based on experimental data demonstrating that hyperbaric oxygen therapy stimulates colonic stem cells and induces mucosal healing [69]. In a prospective case series of 32 patients with medically refractory UC, all patients reported clinical improvement and resolution of hematochezia by the 40th cycle of hyperbaric oxygen therapy [69]. In a subsequent Phase 2A, randomized, double blind, sham-controlled trial of 18 patients with ASUC, a significantly higher proportion of patients treated with hyperbaric oxygen therapy achieved clinical remission at study day five and 10. A larger trial examining the use of hyperbaric oxygen therapy in patients with ASUC is currently underway (ClinicalTrials.gov identifier CT03494764).

Mirakizumab is a p19-directed IL-23 antibody currently in clinical trial for patients with moderate-severe UC. Results through 52 weeks of the Phase 2 trial demonstrated efficacy in induction and maintenance of clinical response [70]. Additional studies are required to determine the optimal dose of mirakizumab.

## 8. Conclusions

Appropriate treatment options for patients with ulcerative colitis vary according to disease severity. The positioning of biologics and small molecules depends on patients’ disease extent and severity, previous medication exposure, and preference. Medication risks and therapeutic benefits should be incorporated in patient discussions to ensure informed decision making.

## Figures and Tables

**Table 1:** Mesalamine formulations.

Trade Name	Formulation	Dose/Frequency Induction	Dose/Frequency Maintenance
Asacol	Oral	2.4–4.8 g daily in three divided doses	1.6 to 2.4 g daily in one-three divided doses
Delzicol	Oral	2.4–4.8 g daily in three divided doses	1.6 g to 2.4 g in one-three divided doses
Lialda	Oral	2.4–4.8 g once daily	2.4–3.6 g once daily
Pentasa	Oral	2–4 g daily in two to four divided doses	1.5–4 g daily in four divided doses
Apriso	Oral	1.5–4.5 g once daily	1.5–3 g daily
Colazal	Oral	2.25 g three times daily	1.5–3 g twice daily
Canasa	Suppository	1 g (1 suppository) once-twice daily	1 g (1 suppository) daily
Rowasa	Enema	4 g (one 60 mL unit) daily once-twice daily	2 to 4 g (30 to 60 mL unit) daily

**Table 2:** Moderate-severe ulcerative colitis therapies.

Therapeutic Class	Mechanism of Action	Formulation
**Anti-TNF agents**	Monoclonal antibodies directed against TNF-alpha, an inflammatory cytokine	
• Infliximab		Intravenous
• Adalimumab		Subcutaneous
• Golimumab		Subcutaneous
**Anti-integrin agents**	Monoclonal antibody directed against α_4_β_7_ cell surface glycoprotein on B and T lymphocytes	
• Vedolizumab		Intravenous
**Anti-interleukin agents**	Monoclonal antibody directed against p40 subunit of IL-12 and IL-23	
• Ustekinumab		Intravenous (1st dose), subcutaneous
**Janus-kinase inhibitors**	Small molecule janus kinase 1 and 3 inhibitor	
• Tofacitinib		Oral
